# Conformation Control of Iminodibenzyl-Based Thermally Activated Delayed Fluorescence Material by Tilted Face-to-Face Alignment With Optimal Distance (tFFO) Design

**DOI:** 10.3389/fchem.2020.00530

**Published:** 2020-08-14

**Authors:** Yu Kusakabe, Yoshimasa Wada, Hiromichi Nakagawa, Katsuyuki Shizu, Hironori Kaji

**Affiliations:** Institute for Chemical Research, Kyoto University, Kyoto, Japan

**Keywords:** organic light-emitting diodes, thermally activated delayed fluorescence, molecular conformation, reverse intersystem crossing, dual emission

## Abstract

In organic light-emitting diodes (OLEDs), all triplet excitons can be harvested as light via reverse intersystem crossing (RISC) based on thermally activated delayed fluorescence (TADF) emitters. To realize efficient TADF, RISC should be fast. Thus, to accomplish rapid RISC, in the present study, a novel TADF emitter, namely, TpIBT-tFFO, was reported. TpIBT-tFFO was compared with IB-TRZ, which contains the same electron donor and acceptor segments, specifically iminodibenzyl and triazine moieties. TpIBT-tFFO is based on a recently proposed molecular design strategy called *tilted face-to-face alignment with optimal distance* (tFFO), whereas IB-TRZ is a conventional through-bond type molecule. According to quantum chemical calculations, a very large RISC rate constant, *k*_RISC_, was expected for TpIBT-tFFO because not only the lowest triplet state but also the second lowest triplet state were close to the lowest excited singlet state, as designed in the tFFO strategy. IB-TRZ has two different conformers, leading to dual emission. Conversely, owing to excellent packing, the conformation was fixed to one in the tFFO system, resulting in single-peaked emission for TpIBT-tFFO. TpIBT-tFFO displayed TADF type behavior and afforded higher photoluminescence quantum yield (PLQY) compared to IB-TRZ. The *k*_RISC_ of TpIBT-tFFO was determined at 6.9 × 10^6^ s^−1^, which is one of the highest values among molecules composed of only H, C, and N atoms. The external quantum efficiency of the TpIBT-tFFO-based OLED was much higher than that of the IB-TRZ-based one. The present study confirms the effectiveness of the tFFO design to realize rapid RISC. The tFFO-based emitters were found to exhibit an additional feature, enabling the control of the molecular conformations of the donor and/or acceptor segments.

## Introduction

Emitting materials for organic light-emitting diodes (OLEDs) have, in recent years, been actively investigated to improve the utilization of triplet excitons. Significant efforts have been devoted to the development of thermally activated delayed fluorescence (TADF) materials (Yang et al., 2017), as they have been demonstrated to convert 100% of both singlet and triplet excitons into light in the absence of any metal atoms in their molecular structures (Uoyama et al., 2012; Kaji et al., 2015; Lin et al., 2016). Hence, TADF materials are expected to be widely applied as alternative emitters to the conventional fluorescence and phosphorescence materials. To realize efficient TADF, the reverse intersystem crossing (RISC) from the lowest triplet state (T_1_) to the lowest excited singlet state (S_1_) should be fast. Basically, effective RISC is expected by minimizing the energy difference between S_1_ and T_1_, namely, Δ*E*_ST_. This can be realized simply by splitting the distribution of the highest occupied molecular orbital (HOMO) and the lowest unoccupied molecular orbital (LUMO), in most cases resulting in charge transfer (CT) type S_1_ and T_1_ (^1^CT and ^3^CT, respectively; Endo et al., 2011). Consequently, many of the reported TADF materials are composed of donor and acceptor moieties, i.e., D-A type molecules (Yang et al., 2017). Nevertheless, small Δ*E*_ST_ is not sufficient to achieve a very large RISC rate constant, *k*_RISC_, typically >10^6^ s^−1^. Notably, incorporation of locally excited triplet states (^3^LE) close in energy to the ^1^CT and ^3^CT states is one of the most promising approaches to accelerate RISC (Dias et al., 2016; Etherington et al., 2016; Gibson et al., 2016; Marian, 2016; Hosokai et al., 2017; Lyskov and Marian, 2017; Samanta et al., 2017; Noda et al., 2018). We have recently proposed a new molecular design principle, namely, *tilted face-to-face alignment with optimal distance* (tFFO) (Wada et al., 2020). In the tFFO molecular design, the degeneration of the three energy levels, i.e., ^1^CT, ^3^CT, and ^3^LE, can be realized with a significant spin–orbit coupling matrix element value (SOCMEV) between the singlet and triplet states, resulting in efficient RISC. The energy level matching of the three levels can be accomplished by optimizing the distance between the electron donor and acceptor fragments (*d*_DA_). Moreover, SOCMEV can be enhanced by tilting the donor and acceptor fragments from the completely parallel face-to-face alignment. On the basis of the tFFO concept, we developed a through-space type molecule, namely, TpAT-tFFO, which was demonstrated to realize very large *k*_RISC_ > 10^7^ s^−1^, despite being composed of only H, C, and N atoms.

**Figure 1:**
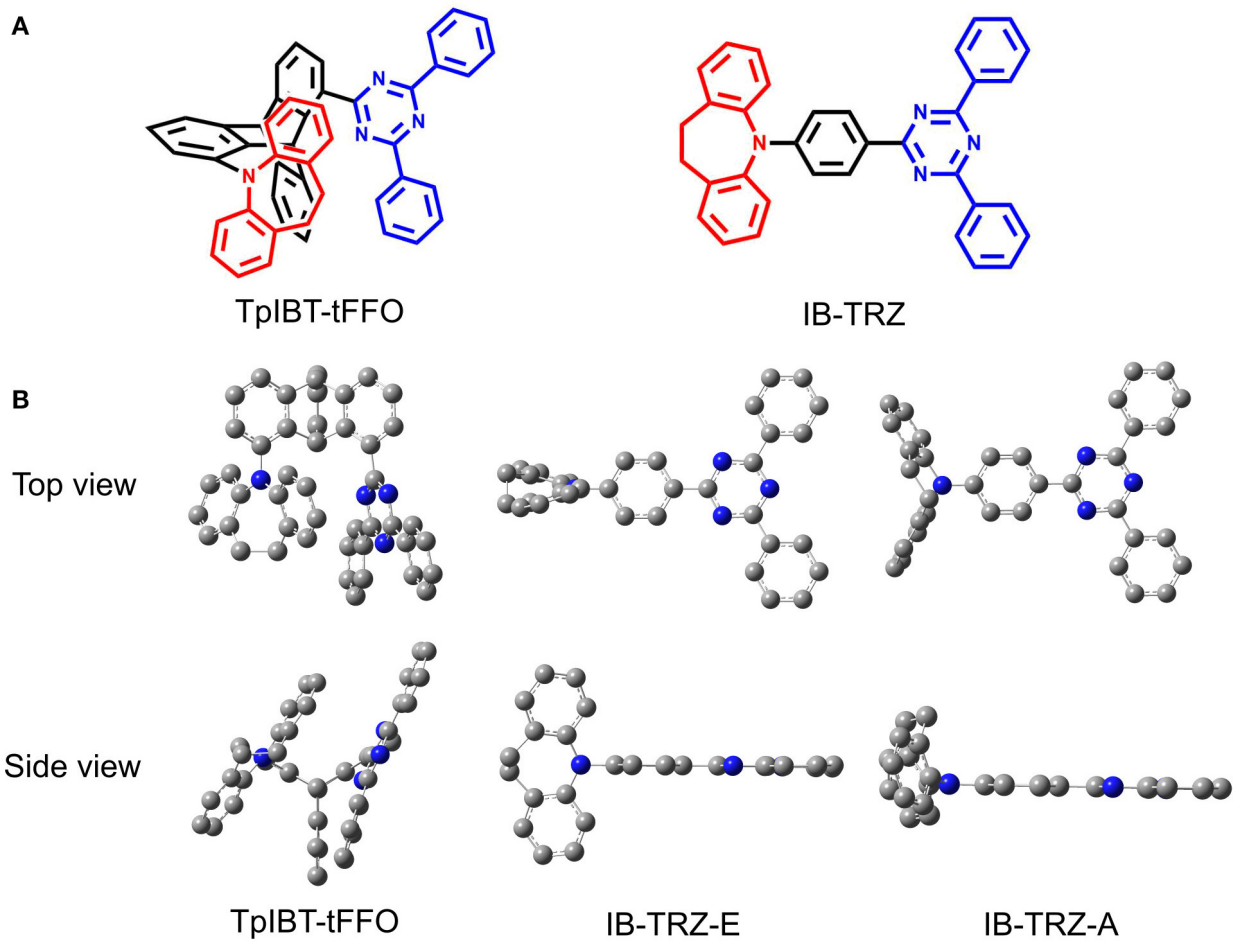
**(A)** Chemical structures of TpIBT-tFFO and IB-TRZ. **(B)** DFT-optimized geometries of TpIBT-tFFO and IB-TRZ.

In the present study, we report another tFFO-based molecule possessing a triptycene (Tp) scaffold, TpIBT-tFFO (Figure 1A, left), which is composed of 10,11-dihydro-5*H*-dibenzo[*b, f* ]azepine (iminodibenzyl, IB) and 2,4-diphenyl-1,3,5-triazine (T) as the donor and acceptor, respectively. For comparison, a molecule comprising IB and T units, IB-TRZ (Figure 1A, right), which was first reported by Huang et al. (2019), was also synthesized as a conventional through-bond D-A type molecule for comparison. As shown later, IB-TRZ has two conformational isomers (Figure 1B), resulting in dual emission. Kukhta et al. first reported on iminodibenzyl-containing TADF molecules, providing evidence for the existence of two conformers (Kukhta et al., 2018). On the other hand, excellent packing in the tFFO systems can fix the D and A conformations, leading to single-peaked emission for TpIBT-tFFO, which is an additional intriguing feature of tFFO molecular design. A large *k*_RISC_ of 6.9 × 10^6^ s^−1^ was obtained for TpIBT-tFFO, which is one of the highest *k*_RISC_ values for molecules composed of only H, C, and N atoms (The highest reported value is also for a tFFO-based molecule; Wada et al., 2020).

## Results and Discussion

The details concerning the synthesis and characterization of both TpIBT-tFFO and IB-TRZ are provided in the Supplementary Material.

### Calculations

**Figure 2:**
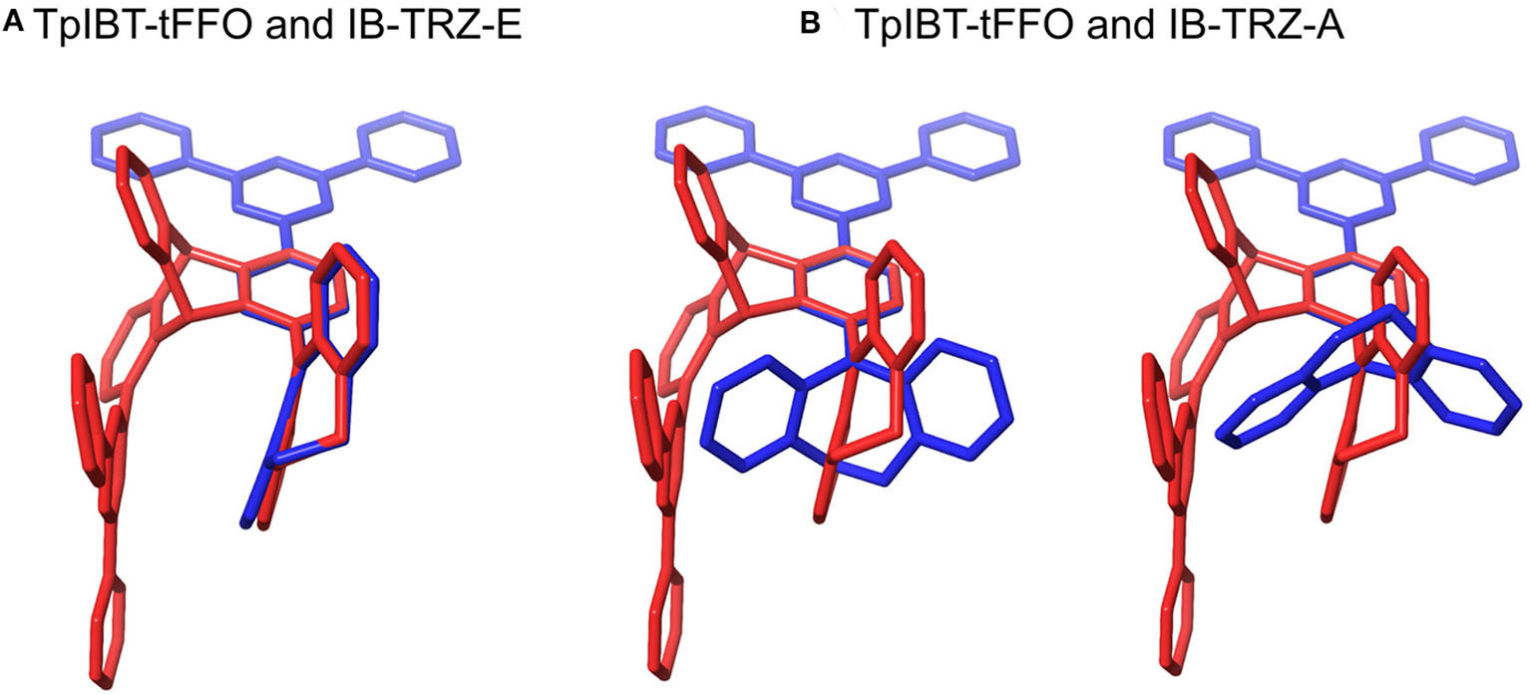
Overlap views of **(A)** TpIBT-tFFO (red) and IB-TRZ-E (blue) as well as **(B)** TpIBT-tFFO (red) and IB-TRZ-A (blue). All structures are DFT-optimized.

**Figure 3:**
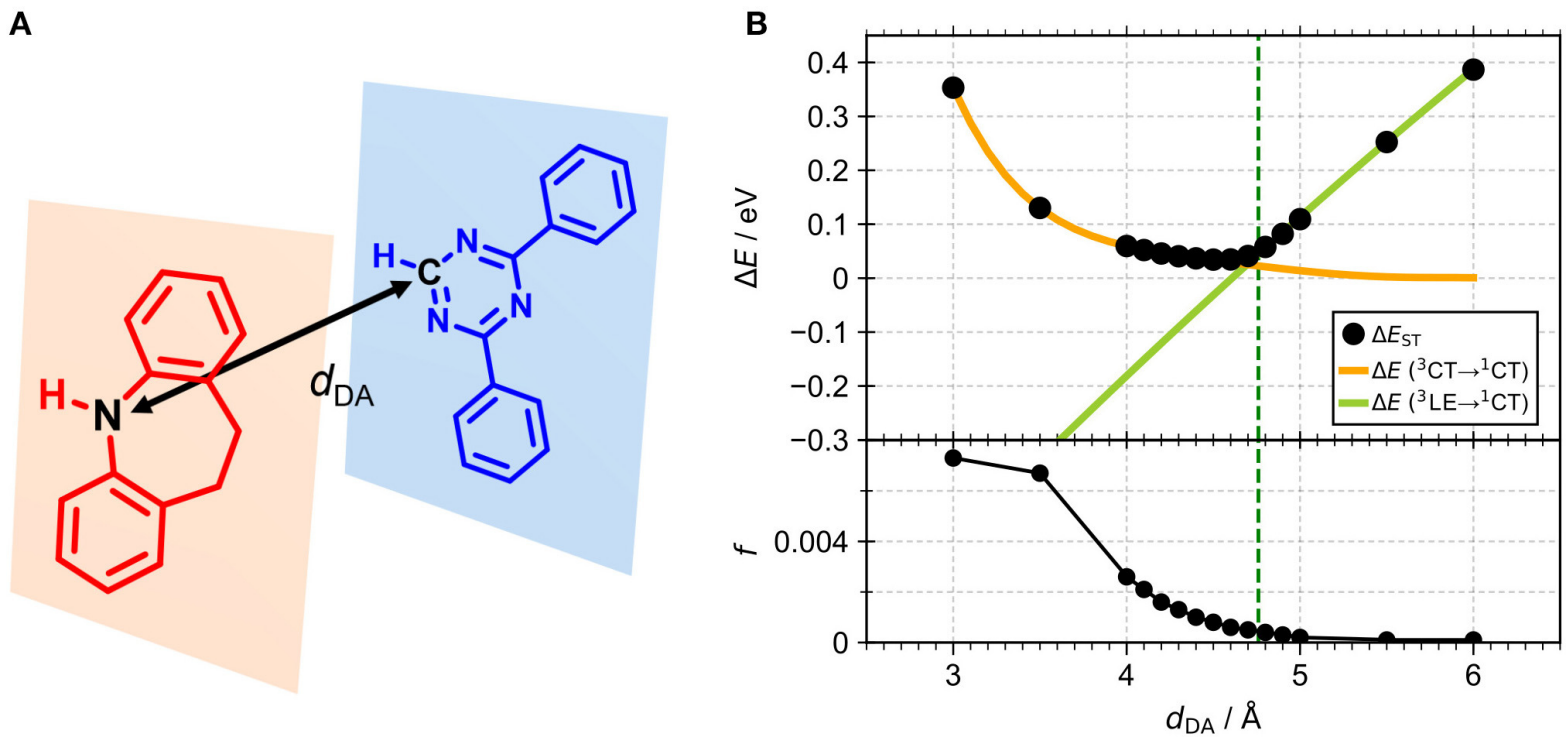
**(A)** The structures of the IB donor and T acceptor. **(B)** Calculated energy differences, Δ*E*_ST_, Δ*E* (^3^CT → ^1^CT) and Δ*E* (^3^LE → ^1^CT) (top) and *f*-values (bottom) as a function of *d*_DA_ for the combination of IB and T. Green dashed line indicates *d*_DA_ of TpIBT-tFFO (4.76 Å).

We performed density functional theory (DFT) at the B3LYP/6-31G(d) level to optimize the ground state (S_0_) geometries utilizing Gaussian 16. Figure 1B demonstrates the optimized molecular geometries of TpIBT-tFFO and IB-TRZ. For IB-TRZ, two energetically stable conformers were found depending on the initial conformations. One is a quasi-equatorial conformation, named IB-TRZ-E, where the donor and acceptor fragments are perpendicular to each other, while the other is quasi-axial, denoted as IB-TRZ-A. The total energy of IB-TRZ-E was 0.324 eV larger than that of IB-TRZ-A. The donor and acceptor segments of IB-TRZ-A are also nearly perpendicular to each other, however, in a different direction. The nitrogen atom in the IB units is not sp^3^- but sp^2^-hybridized in both conformers. The nitrogen and the directly bonded three carbon atoms form not a pyramidal, but a planar structure. The donor of IB-TRZ-A, IB, exhibits a butterfly shape bended in the middle (Figure 2), while TpIBT-tFFO has only one geometry, corresponding to an equatorial conformation. To visually demonstrate the difference between TpIBT-tFFO and IB-TRZ-E (or -A), the overlap views of TpIBT-tFFO and IB-TRZ-E as well as of TpIBT-tFFO and IB-TRZ-A are illustrated in Figures 2A,B, respectively. As can be seen, the IB units of TpIBT-tFFO and IB-TRZ-E are well overlapped, while the IB moieties of TpIBT-tFFO and IB-TRZ-A are significantly deviated. The nearest inter-atomic distance between IB in the axial conformation and T is too close; thus, the axial conformation in the tFFO type molecule is physically impossible, as evidenced in Figure 2B. For the optimized structure, DFT and time-dependent DFT (TD-DFT) calculations at the LC-ωPBE/6-31+G(d) level were also performed to estimate the energy levels of the HOMO, LUMO, and excited states more adequately. Here, ω is the optimized range-separation parameter of the LC-ωPBE functional, employed to precisely predict the excited state energies of the TADF materials. The optimized ω for TpIBT-tFFO was determined according to a previously reported method (Sun et al., 2015). Table 1 summarizes the calculation results of the HOMO and LUMO distributions, energy levels of the HOMO, LUMO, S_1_, and T_1_ as well as Δ*E*_ST_ for TpIBT-tFFO, IB-TRZ-E, and IB-TRZ-A. The HOMO and LUMO distributions were significantly different between IB-TRZ-E and IB-TRZ-A, in which the central phenylene ring was included in the LUMO and HOMO, respectively. The HOMO/LUMO energy levels were −6.70/−0.99 and −6.91/−0.75 eV for IB-TRZ-E and IB-TRZ-A, correspondingly. Furthermore, the S_1_ and T_1_ energy levels and the resulting Δ*E*_ST_ exhibited large differences (the Δ*E*_ST_s of IB-TRZ-E and IB-TRZ-A were 0.30 and 0.81 eV, respectively). In the case of TpIBT-tFFO, HOMO and LUMO were well spatially separated. On the basis of the calculated Δ*E*_ST_ of 0.032 eV, TpIBT-tFFO is expected to be a promising TADF emitter. More importantly, the T_2_ state lies closely above the S_1_ state (specifically, 0.0006 eV above). We also theoretically calculated the energy difference, Δ*E*_ST_, those from ^3^CT to ^1^CT, Δ*E* (^3^CT → ^1^CT), and from ^3^LE to ^1^CT, Δ*E* (^3^LE → ^1^CT) as a function of the distance between the donor (IB) and acceptor (T), *d*_DA_ (Figure 3). The calculations for the oscillator strength (*f*) are also shown. Here, *d*_DA_ is defined as the distance between the nitrogen atom in IB and the carbon atom in T. Excellent energy level matching of ^1^CT, ^3^CT, and ^3^LE, is achieved at approximately *d*_DA_ = 4.5–5 Å. Using a triptycene scaffold, we realized the *d*_DA_ of 4.76 Å, which resulted in excellent energy matching of the three states within 0.04 eV. We also carried out SOCMEV calculations employing the ADF2018 package based on the Brédas' calculation level (Samanta et al., 2017). The LCY-ωPBE functional as well as Slater-type all-electron TZP were used with the range parameter, γ (Akinaga and Ten-no, 2008), which was optimized at 0.21. TpIBT-tFFO exhibited SOCMEVs of 0.02 and 0.46 between S_1_ and T_1_, and between S_1_ and T_2_, respectively (Table S1). The SOCMEV between S_1_ and T_2_ was large owing to the tilted IB and T alignments created by the Tp scaffold, where the tilt angle of TpIBT-tFFO was ~20°.

**Table 1:** Distributions of the HOMO and LUMO, energy levels of HOMO, LUMO, Δ*E*_ST_, S_1_, and T_1_ of TpIBT-tFFO and IB-TRZ calculated at LC-ωPBE/6-31+G(d).

**Emitter**		**HOMO Distribution**	**LUMO Distribution**	**HOMO (eV)**	**LUMO (eV)**	**S_**1**_ (eV)**	**T_**1**_ (eV)**	**Δ*E*_**ST**_ (eV)**
TpIBT-tFFO		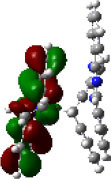	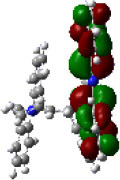	−6.44	−0.78	3.18	3.15	0.03
IB-TRZ	IB-TRZ-E	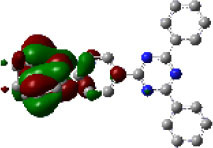	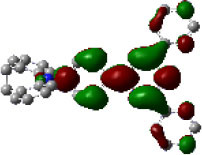	−6.70	−0.99	3.12	2.83	0.30
	IB-TRZ-A	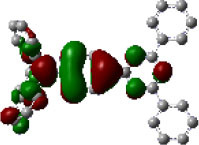	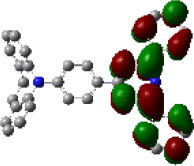	−6.91	−0.75	3.51	2.70	0.81

### Thermal and Photophysical Properties

**Figure 4:**
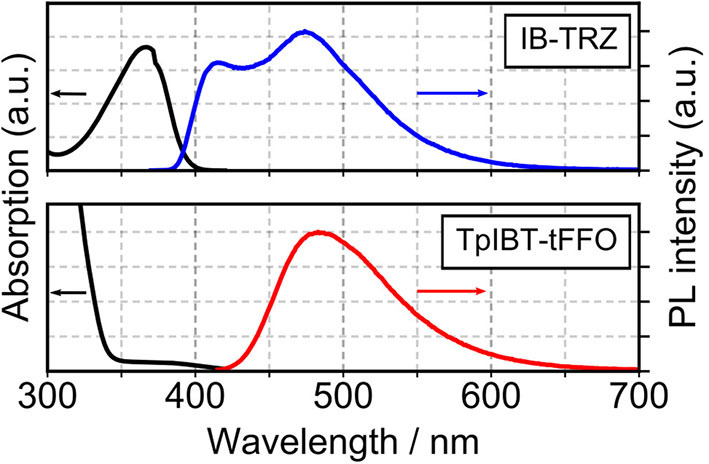
UV-vis absorption (black) of TpIBT-tFFO and IB-TRZ and the PL spectra of TpIBT-tFFO (red) and IB-TRZ (blue) in a toluene solution (10^−4^ M).

**Figure 5:**
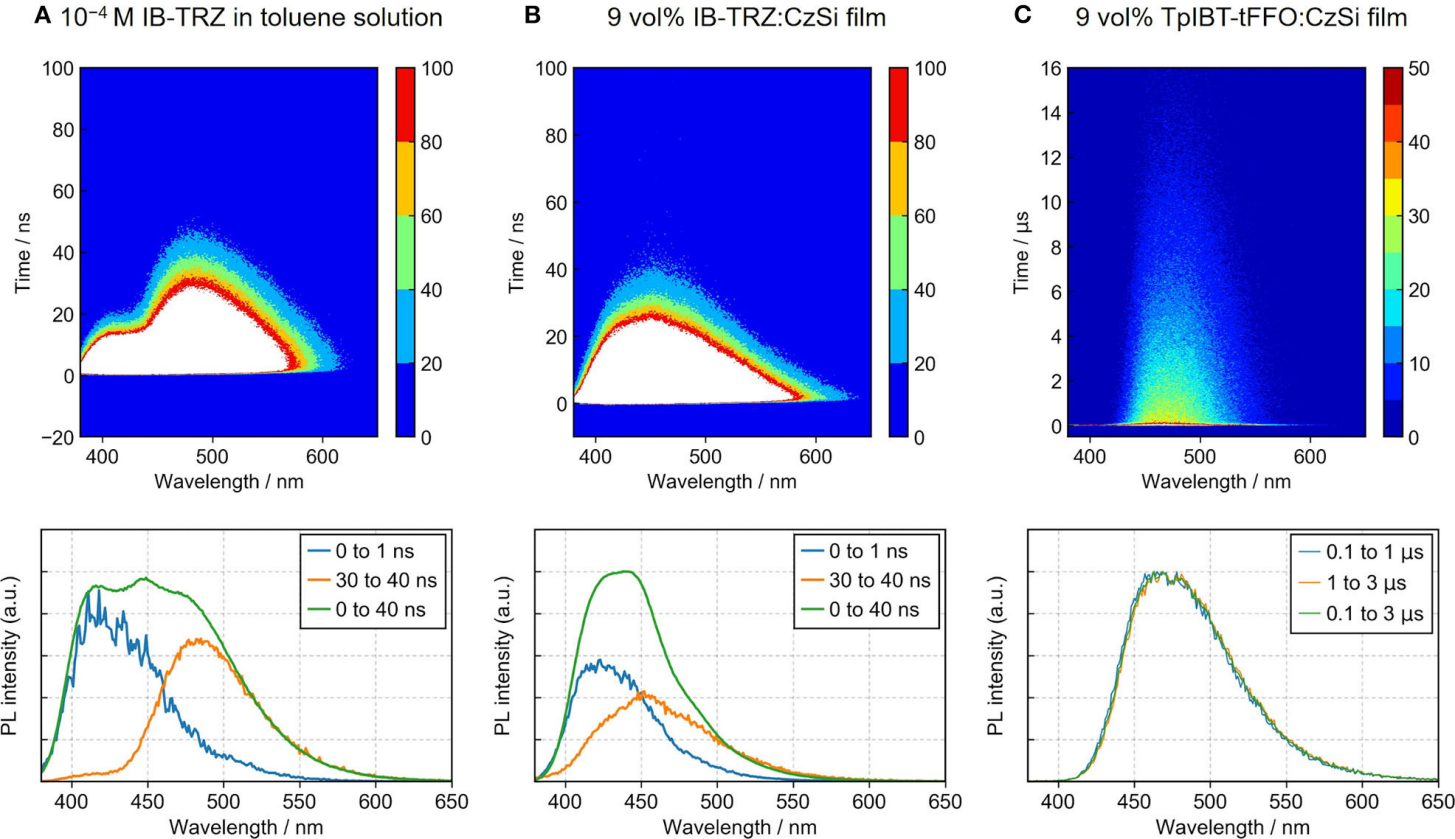
Time-dependent PL measurements of **(A)** IB-TRZ in a toluene solution, **(B)** 9 vol% IB-TRZ:CzSi film, and **(C)** 9 vol% TpIBT-tFFO:CzSi film. Contour plots of the PL spectra as a function of time (top) and time-dependent PL spectra (bottom) are provided.

The TGA curves of both TpIBT-tFFO and IB-TRZ are provided in Figure S3. The decomposition temperatures, *T*_d_s, of TpIBT-tFFO and IB-TRZ were determined at 396 and 386°C, respectively, from the 5% weight loss point of the TGA curve. Figure 4 shows the ultraviolet-visible (UV-vis) absorption and photoluminescence (PL) spectra of IB-TRZ and TpIBT-tFFO in 10^−4^ M toluene solutions. IB-TRZ exhibited dual emission with the first and second peak maximum wavelength (λ_MAX_) of 417 and 474 nm, respectively, while TpIBT-tFFO displayed single-peaked emission with λ_MAX_ of 485 nm. Considering the above calculation results, the emissions of IB-TRZ at shorter and longer wavelengths were rationally assigned to IB-TRZ-A and IB-TRZ-E, correspondingly. The assignment was further confirmed by transient PL experiments described below. To investigate the TADF performance of IB-TRZ and TpIBT-tFFO, we carried out PL quantum yield (PLQY) measurements for the toluene solution under O_2_ or Ar bubbling for 15 min. In the case of IB-TRZ, the PLQY differed: 11.0% (O_2_) and 26.1% (Ar). On the other hand, the PLQY of TpIBT-tFFO in the toluene solution considerably increased from 1.1% (O_2_) to 52.4% (Ar). This is a typical feature of tFFO type molecules, which exhibit significantly larger *k*_ISC_ and *k*_RISC_ than $k_{\text{r}}^{\text{S}}$ and $k_{\text{nr}}^{\text{S}}$ (Wada et al., 2020). Here, *k*_ISC_, $k_{\text{r}}^{\text{S}}$, and $k_{\text{nr}}^{\text{S}}$ indicate the rate constants of intersystem crossing, radiative decay from S_1_, and non-radiative decay from S_1_, respectively. Considering that O_2_ molecules behave as triplet quenchers, these outcomes indicate that a triplet state was strongly involved in the TpIBT-tFFO emission process. To determine Δ*E*_ST_ experimentally, we performed a PL measurement at 77 K (Figure S4). In most cases, Δ*E*_ST_ of the TADF materials was established based on the energy difference between the onset of the PL (fluorescence) spectrum at RT and that of the PL (phosphorescence) spectrum at low temperature. According to this procedure, we obtained a negative Δ*E*_ST_ of −0.13 eV for TpIBT-tFFO. Previously, negative Δ*E*_ST_ values for TADF molecules has been reported in some articles (Di et al., 2017; Kim et al., 2018; Braveenth et al., 2019); however, the Arrhenius plots of *k*_ISC_ and *k*_RISC_ provided positive Δ*E*_ST_, 0.0061 eV (the difference of activation energies for RISC and ISC, see Table 4 below), although the value was obtained for solid film sample. We speculate that the negative value obtained from the above procedure might be a consequence of smaller structural relaxations after vertical excitation from S_0_ at 77 K than at RT. Another possible origin would be different S_0_ structures between the vertical transitions from S_1_ and from T_1_. Thus, we determined Δ*E*_ST_ based on the energy difference between the onset of the steady state PL spectrum at 77 K (corresponding to S_1_, the spectrum also includes phosphorescence but the onset is determined by S_1_.) and that of the integrated PL spectrum from 1 to 10 ms at 77 K (corresponding to T_1_). We believe that this is a more reasonable approach to establish Δ*E*_ST_ experimentally. The Δ*E*_ST_ values of IB-TRZ and TpIBT-tFFO were determined at 0.41 and 0.0076 eV, respectively (Table 2). These experimentally-determined values reasonably reflect the calculated ones (Table 1) and the value estimated from the Arrhenius plots. To understand the dual emissions of IB-TRZ, we performed a transient PL measurement for the solution. Figure S5C shows the transient PL decay curves of IB-TRZ observed at 400 and 500 nm, which were used to monitor the emission from IB-TRZ-A and IB-TRZ-E, respectively. For IB-TRZ, the PL decay curves with the lifetime of prompt fluorescence, τ_p_, of 3.3 and 10.9 ns were observed at 400 and 500 nm, respectively. Table 3 summarizes the obtained photophysical parameters. Time-dependent PL spectra evidently revealed that IB-TRZ exhibits two different emissions as discussed later (Figures 5A,B). Moreover, no clear delayed fluorescence with the μs-order lifetime was found in IB-TRZ. In the case of TpIBT-tFFO, in addition to normal (prompt) fluorescence with a lifetime of 11.7 ns, delayed fluorescence with a lifetime of 2.5 μs was also clearly observed following Ar gas bubbling (Figure S5D). The lifetime of the prompt component was in good agreement with that of IB-TRZ-E (10.9 ns). The emission of TpIBT-tFFO was primarily composed of the delayed component, which disappeared after O_2_ gas bubbling (Figure S5B). From the PLQY and transient PL measurements of TpIBT-tFFO, 93.6% of the total PLQY was attributed to the delayed emission. These results indicate that TpIBT-tFFO is a TADF-active emitter. The *k*_ISC_ and *k*_RISC_ values of TpIBT-tFFO were experimentally determined at 7.4 × 10^7^ s^−1^ and 6.9 × 10^6^ s^−1^, respectively, utilizing our previously reported method (Wada et al., 2020). The large *k*_RISC_ value is by virtue of the tFFO design.

**Table 2:** Experimentally obtained thermal properties, energy levels of HOMO, LUMO, *E*_g_, S_1_, T_1_, and Δ*E*_ST_ for TpIBT-tFFO and IB-TRZ.

**Emitter**	***T*_**d**_ (^**°**^C) ^a^**	**HOMO (eV)^b^**	**LUMO (eV)^c^**	***E*_***g***_ (eV)^d^^,^^e^**	**S_**1**_ (eV)^d^**	**T_**1**_ (eV)^d^**	**Δ*E*_***ST***_ (eV)^d^^,^^i^**
TpIBT-tFFO	396	−5.73	−2.95	2.78	3.08^f^ (2.94^g^)	3.07^h^	0.0076
IB-TRZ	386	−5.87	−2.80	3.07	3.17^f^ (3.24^g^)	2.76^h^	0.41

a Determined from the 5% weight loss point of the TGA curve. Determined from

b the onset of photoelectron spectrum in air and

c *the equation LUMO = E_g_ + HOMO*.

d *Measured in a toluene solution with the emitter concentration of 10^−4^ M*.

e *Determined from the onset of the UV-vis absorption spectrum*.

f *Determined from the onset of the steady state PL spectrum at 77 K*.

g Determined from the onset of the PL spectrum at room temperature. Determined from

h the onset of the integrated PL spectrum from 1 to 10 ms at 77 K, and

i *the equation ΔE_ST_ = S_1_–T_1_*.

**Table 3:** Photophysical parameters of λ_MAX_, Φ_PL_, τ_p_, τ_d_, *k*_ISC_, and *k*_RISC_ for TpIBT-tFFO and IB-TRZ.

**Emitter**	**State**	**λ_**MAX**_ (nm)^c^**	**Φ_**PL**_ (%)**	**τ_**p**_ (ns)**	**τ_**d**_ (μs)**	***k*_**ISC**_ (10^**7**^ s^**−1**^)^n^**	***k*_**RISC**_ (10^**6**^ s^**−1**^)^n^**
TpIBT-tFFO	sol^a^	485	52.4^d^^,^^e^(1.1^d^^,^^f^)	11.7^j^	2.5^j^	7.4	6.9
	film^b^	477	71.4^g^^,^^h^	24.5^k^	6.7^k^	3.6	2.3
IB-TRZ	sol^a^	418, 485	26.1^d^^,^^e^ (11.0^d^^,^^f^)	3.3^l^, 10.9^m^	–	–	–
	film^b^	423, 452	23.5^g^^,^^i^	2.9^l^, 5.2^m^	–	–	–

Measured ^a^in a toluene solution with the emitter concentration of 10^−4^ M and

b *from the 9 vol% emitter:CzSi film*.

c *Determined from the time-dependent spectra for IB-TRZ*.

*Measured ^d^with the excitation wavelength of 365 nm*;

e *after 15 min of Ar bubbling*;

f *after 15 min of O_2_ bubbling*;

g *under N_2_ flow*;

h *at the excitation wavelength of 340 nm*;

i *at the excitation wavelength of 350 nm*;

j *at the observed wavelength of 485 nm*;

k *at the observed wavelength of 477 nm*;

l *at the observed wavelength of 400 nm*;

m *at the observed wavelength of 500 nm*.

n *Calculated according to a previously reported method (Wada et al., 2020)*.

To investigate the TADF characteristics of IB-TRZ and TpIBT-tFFO in amorphous films, IB-TRZ and TpIBT-tFFO were doped into the host matrix. For this purpose, 9-(4-(*tert*-butyl)phenyl)-3,6-bis(triphenylsilyl)-9*H*-carbazole (CzSi) with high T_1_ energy of 3.02 eV was used as the host to confine the triplet energies of IB-TRZ and TpIBT-tFFO. Figure S6A illustrates the PL spectrum of the 9 vol% IB-TRZ:CzSi film. Unlike the spectrum in solution (Figure S5A), seemingly one peak top at approximately 421 nm was observed. The transient PL experiment (Figure S6C) demonstrated different τ_p_ of 2.9 and 5.2 ns at 400 and 500 nm, respectively. The time-dependent PL spectrum shown in Figure 5B confirmed that the PL spectrum was composed of two different emissions. The distinct two peak tops were not found because the emission wavelength with longer τ_p_ in the film came closer to that with shorter τ_p_ (452 and 423 nm, respectively), compared to the case in solution (485 and 418 nm, respectively). The value of longer τ_p_ in the film (5.2 ns) was also closer to that of shorter τ_p_ (2.9 ns) compared to the solution case (10.9 and 3.3 ns, respectively). These results indicate that the component with the emission wavelength of 452 nm in the film originated from IB-TRZ with the intermediate structure between quasi-equatorial and quasi-axial conformers.

**Figure 6:**
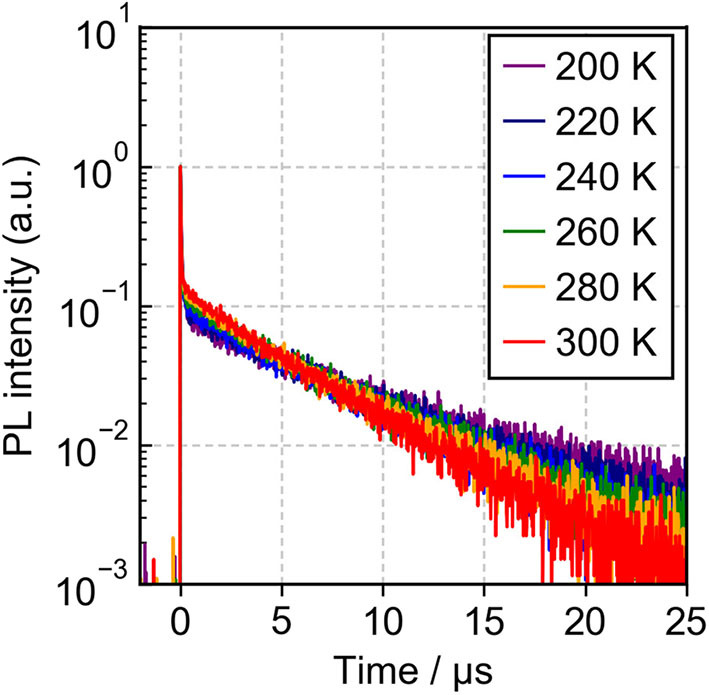
PL decay curves of the 9 vol% TpIBT-tFFO:CzSi film observed at 470 nm from 200 to 300 K.

Meanwhile, the 9 vol%TpIBT-tFFO:CzSi film exhibited a PL spectrum with λ_MAX_ of 477 nm (Figure S6B), which was composed of only one emission, as evidenced by the transient PL experiment demonstrated in Figure 5C. The emission wavelength of 477 nm was also consistent with that in the solution. Analogously to the solution case, two clear exponential decays with the lifetimes of 24.5 ns and 6.7 μs were observed (Figure S6D), and the emission spectra in TpIBT-tFFO did not change with time (Figure 5C), confirming the TADF character of TpIBT-tFFO. These results indicate that the molecular conformation can be effectively controlled in the tFFO system, resulting in single-peaked emission. Under N_2_ gas flow, the PLQY of the 9 vol% TpIBT-tFFO:CzSi film was relatively high (71.4%) compared with that of the 9 vol% IB-TRZ:CzSi film (23.5%). The higher PLQY is a result of the availability of efficient RISC in TpIBT-tFFO. We also carried out temperature-dependent transient PL measurements to examine the energy level matching of the excited states in TpIBT-tFFO (Figure 6). From the Arrhenius plots of *k*_ISC_ and *k*_RISC_, activation energies for ISC (*E*_ISC_) and RISC (*E*_RISC_) were determined at 0.0305 and 0.0366 eV, respectively. Combined with the calculation results described above, we established the experimental energy differences between S_1_ and T_2_ [Δ*E* (S_1_↔T_2_)] as well as between T_1_ and T_2_ [Δ*E* (T_1_↔T_2_)] (Table 4). Excellent energy level matching of the three states was successfully realized in TpIBT-tFFO.

**Table 4:** Energy differences of T_1_ and T_2_ [Δ*E* (T_1_↔T_2_)] and S_1_ and T_2_[Δ*E* (S_1_↔T_2_)] for TpIBT-tFFO obtained experimentally and via a DFT calculation.

**Energy difference**	**Experiment**	**Calculation**
Δ*E* (S_1_↔T_2_) (eV)	0.0305	0.0006
Δ*E* (T_1_↔T_2_) (eV)	0.0366	0.0326

### Electroluminescence Performance

**Figure 7:**
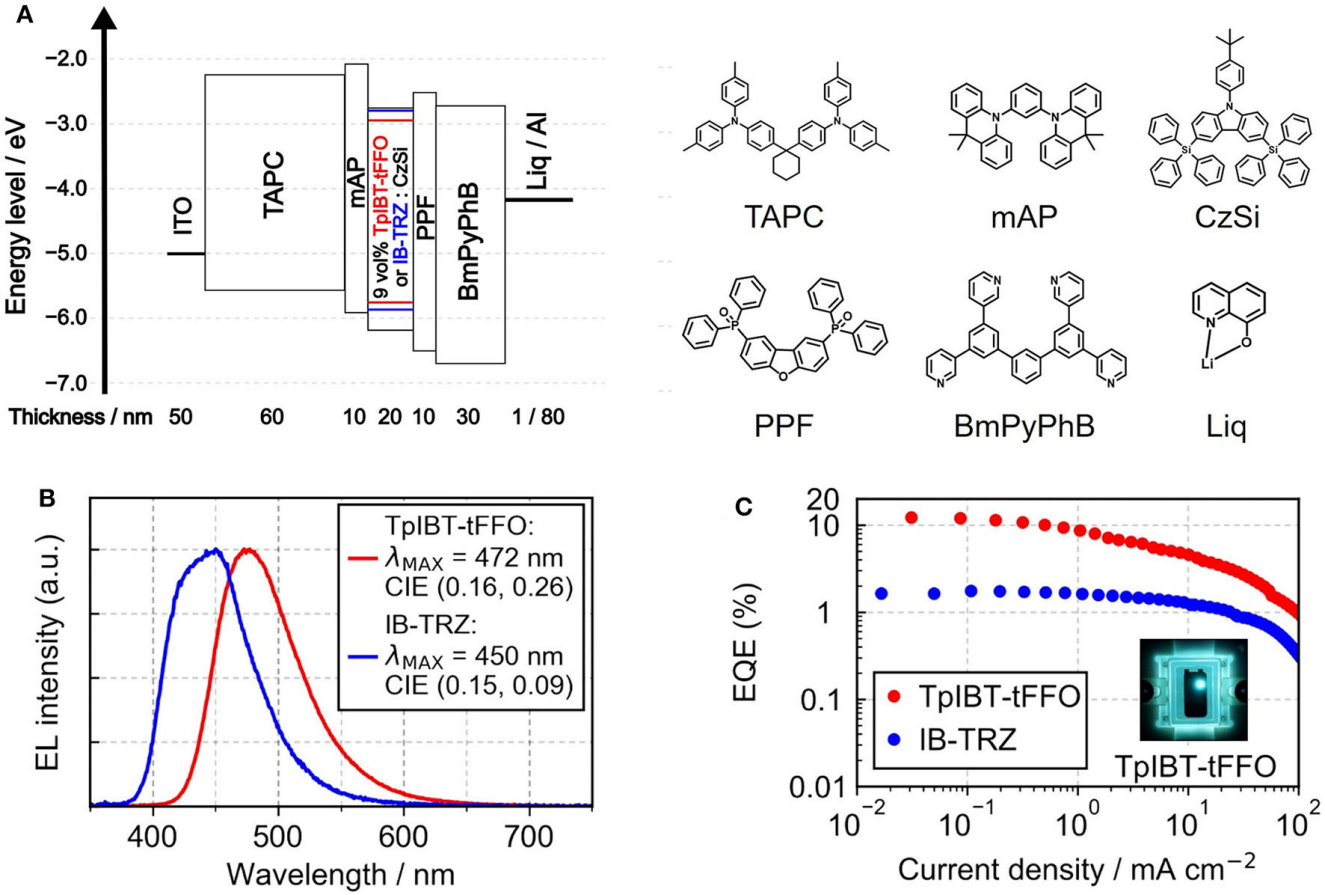
**(A)** Device structures of the TpIBT-tFFO- and IB-TRZ-based OLEDs and the utilized materials. The HOMO/LUMO energy levels of TpIBT-tFFO and IB-TRZ are depicted in red and blue lines, respectively. **(B)** EL spectra of TpIBT-tFFO- and IB-TRZ-based OLEDs. **(C)** EQEs of TpIBT-tFFO- and IB-TRZ-based OLEDs as a function of current density.

From the photophysical observations, we established that TpIBT-tFFO is a promising TADF emitter exhibiting high triplet-to-singlet conversion efficiency. This motivated us to fabricate TpIBT-tFFO-based OLED. For comparison, we also fabricated IB-TRZ-based OLED. Figure 7A shows the device structure, indium tin oxide (ITO) (50 nm)/1,1-bis[4-[*N, N*-di(p-tolyl)amino]phenyl]cyclohexane (TAPC) (60 nm)/1,3-bis(9,9-dimethylacridin-10(9*H*)-yl)benzene (mAP) (10 nm)/9 vol% IB-TRZ or TpIBT-tFFO:CzSi (20 nm)/2,8-bis(diphenylphosphoryl)dibenzo[b,d]furan (PPF) (10 nm)/1,3-bis[3,5-di(pyridin-3-yl)phenyl]benzene (BmPyPhB) (30 nm)/lithium quinolin-8-olate (Liq) (1 nm)/Al (80 nm). Figures 7B,C illustrate the EL spectra and external quantum efficiency (EQE)-current density characteristics of the IB-TRZ- and TpIBT-tFFO-based OLEDs. A summary of the device performances is provided in Table 5. The EL spectra of the OLEDs were substantially identical to the corresponding PL spectra, with λ_MAX_ at 450 and 472 nm for IB-TRZ and TpIBT-tFFO, respectively. The TpIBT-tFFO-based OLED exhibited maximum EQE (EQE_MAX_) of 12.2%, which is approximately seven times higher than that of the IB-TRZ-based OLED (EQE_MAX_ = 1.8%). This remarkable improvement of EQE can be attributed to the efficient TADF of TpIBT-tFFO.

**Table 5:** OLED performances of TpIBT-tFFO and IB-TRZ.

**Emitter**	**λ_**MAX**_ (nm)**	**CIE (*x*, *y*)**	**EQE_**MAX**_ (%)**	***V*_**on**_ (V)^a^**	**PE_**MAX**_ (lm W^**−1**^)**	**CE_**MAX**_ (cd A^**−1**^)**
**TpIBT-tFFO**	472	(0.16, 0.26)	12.2	3.2	20.8	22.1
**IB-TRZ**	450	(0.15, 0.09)	1.8	3.8	1.3	1.4

a *Determined from the first voltage exceeding 1 cd m^−2^*.

## Conclusion

In summary, in the present study, we developed a tFFO-based TADF material, i.e., TpIBT-tFFO, and compared it with IB-TRZ. Both materials contain an iminodibenzyl moiety as an electron donor unit. The TD-DFT calculations demonstrated that not only T_1_ but also T_2_ were lying close to the S_1_ state in TpIBT-tFFO, as we designed for. From photophysical properties, IB-TRZ had two different conformers, resulting in dual emission. In contrast, the conformation was effectively controlled in the tFFO system, providing a single-peaked emission in TpIBT-tFFO. Furthermore, TpIBT-tFFO exhibited higher PLQY and higher EQE values compared to IB-TRZ. The *k*_RISC_ of TpIBT-tFFO was determined to be 6.9 × 10^6^ s^−1^, which is one of the highest values in TADF materials composed of only H, C, and N atoms, as a consequence of the tFFO strategy.

## Data Availability Statement

All datasets generated for this study are included in the article/Supplementary Material.

## Author Contributions

HK proposed the idea of tFFO and KS, YW, and YK proposed the idea of IBT-based TADF. YW and YK performed quantum chemical calculations and carried out characterization. HN, YW, and YK contributed to the synthesis of the materials. YK fabricated the devices under the supervision of YW. All authors participated in writing the manuscript. YW and HK planned and supervised this study. All authors contributed to the article and approved the submitted version.

## Conflict of Interest

The authors declare that the research was conducted in the absence of any commercial or financial relationships that could be construed as a potential conflict of interest.
