# Supplementary material for: Conformation Control of Iminodibenzyl-Based Thermally Activated Delayed Fluorescence Material by Tilted Face-to-Face Alignment With Optimal Distance (tFFO) Design
Source: Front Chem. 2020 Aug 14;8:530. doi: 10.3389/fchem.2020.00530 (PMC7456890; doi:10.3389/fchem.2020.00530)
Supplement: Supplementary file 1 [file Data_Sheet_1.pdf]

Supplementary Material

General procedure

The DFT, TD-DFT, and SOCMEV calculations

The DFT and TD-DFT calculations were performed at the B3LYP/6-31G(d) and LC-ωPBE/6-31+G(d) levels of theory, respectively, using the Gaussian16 platform. The calculations were conducted in toluene solutions, as implemented in the polarizable continuum model (PCM). SOCMEVs were calculated utilizing the ADF2018 software, in which the LCY-ωPBE functional and Slater-type all-electron TZP were employed. Optimization of the range-separation parameter, ω, for LC-ωPBE was carried out based on a previously described method (Samanta et al., 2017). Determination of γ for the LCY-ωPBE functionals was also conducted in accordance with a previously reported approach (Wada et al., 2020).

Material characterization

^{1}H and ^{13}C NMR spectra were recorded utilizing a Bruker Avance-III spectrometer. Chemical shifts are reported in δ ppm. Residual protons in deuterated solvents were used as internal standards for the ^{1}H NMR experiments. For the ^{13}C NMR spectra, solvent peaks were utilized as internal standards. Atmospheric pressure chemical ionization (APCI) mass spectra were measured with a Bruker micrOTOF-Q II spectrometer.

Thermal properties

Decomposition temperatures (T_d) were determined at 5% weight loss using thermogravimetric analysis (TG-DTA 2000SEα, NETZSCH Japan K.K.).

Photophysical properties

UV-vis absorption and photoluminescence (PL) spectra were measured using a UV-vis spectrophotometer (UV-2600, SHIMAZU, Japan) and a spectrofluorometer (FluoroMax Plus, HORIBA, Japan), respectively. The HOMO energy level was determined utilizing photoelectron yield spectroscopy in air (AC-3, Riken Keiki, Japan). The photoluminescence quantum yield (PLQY) was measured employing an absolute PLQY spectrometer (C9920-02, Hamamatsu Photonics, Japan). The temperature-dependent transient PL measurements were performed using a fluorescence lifetime measurement system (Quantaurus-Tau C11367-01, Hamamatsu Photonics, Japan) equipped with a cryostat (Oxford Instruments, Optistat DN2, UK). For solution measurements, a toluene solution with a concentration of 10^{-4} M was prepared, for which spectrochemical analysis grade toluene was used without further purification (FUJIFILM Wako Pure Chemical Corporation, Japan). O_2 or Ar gas was bubbled for 15 min at an excitation wavelength of 365 nm. In the case of the film samples, measurements were performed under N_2 flow with an excitation wavelength of 350 nm (IB-TRZ) or 340 nm (TpIBT-tFFO).

Device fabrication

The ITO substrates were cleaned prior to deposition by sonication in detergent and H_2O for 10 min (twice), followed by exposure to the steam of 2-propanol for 5 min, and UV-O_3 irradiation for 30 min. All depositions were conducted using a deposition apparatus (SE-4260, ALS Technology, Japan) at ~10^{-4} Pa. The OLEDs were finally attached to getter films to remove oxygen and moisture and sealed with glass caps.

Device characterization

The OLED performance was measured employing an integrating sphere (C9920-12, Hamamatsu Photonics, Japan) and a source meter (2400, Keithley, Japan) under the condition of the forward direction in 200 mV steps.

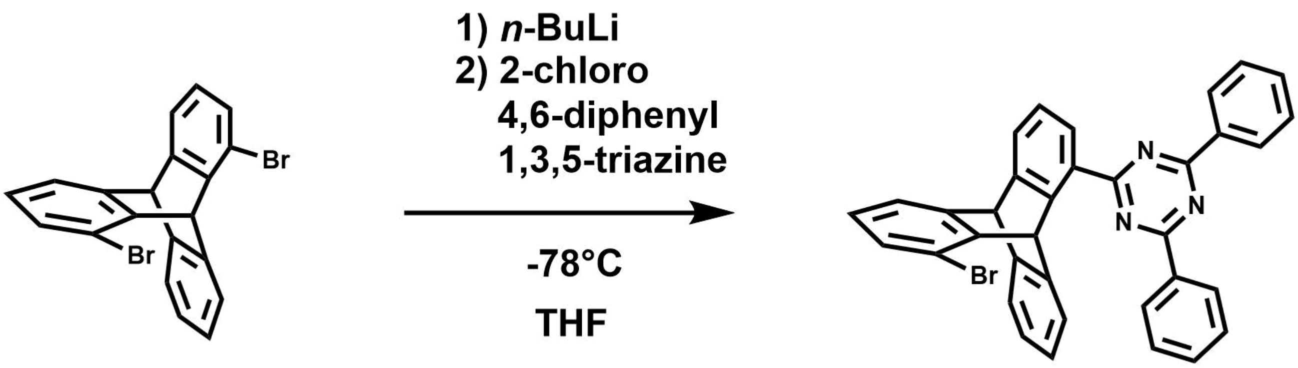

Compound S1

Compound S1: To a solution of 1,8-dibromotriptycene (4.12 g, 10.0 mmol) in 100 mL of anhydrous THF cooled to −78 °C, a 1.6 M solution of *n*-butyllithium in hexanes (7.50 mL, 12.0 mmol) was added dropwise. After stirring for 2 h at −78 °C, 2-chloro-4,6-diphenyl-1,3,5-triazine (3.22 g, 12.0 mmol) in 50 mL of anhydrous THF was added dropwise, and the reaction mixture was slowly warmed up to room temperature. The crude solution was quenched with 1 M aqueous solution of HCl, and the resulting mixture was extracted three times with 50 mL of ethyl acetate. The combined organic layers were further washed with water and brine, and then dried over sodium sulfate. The crude mixture was concentrated under reduced pressure and subsequently purified by silica gel column chromatography using hexane/dichloromethane = 7/3 as the eluent. **Compound S1** was obtained in 69% yield (3.90 g, 6.90 mmol).

^{1}H NMR ($CDCl_3$, δ): 8.90 (d, *J* = 6 Hz, 4H), 8.21 (d, *J* = 6 Hz, 1H), 7.93 (s, 1H), 7.78 (d, *J* = 6 Hz, 1H) 7.68–7.63 (m, 6H), 7.61 (d, *J* = 6.0 Hz, 1H), 7.49 (d, *J* = 6.0 Hz, 1H), 7.32 (d, *J* = 6.0 Hz, 1H), 7.23 (t, *J* = 6.0 Hz, 1H), 7.15 (t, *J* = 6.0 Hz, 1H), 7.10 (t, *J* = 6.0 Hz, 2H), 6.83 (t, *J* = 6.0 Hz, 1H), 5.55 (s, 1H)

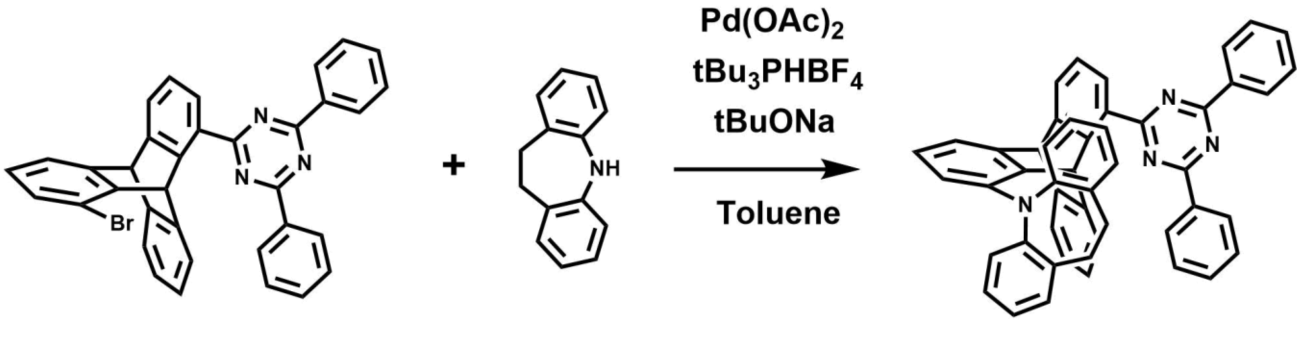

TpIBT-tFFO: Under Ar atmosphere, 20 mL of anhydrous toluene was added to a 50 mL round bottom Schlenk flask containing **Compound S1** (1.13 g, 2.00 mmol), 10,11-dihydro-5*H*-dibenzo[*b*,*f*]azepine (469 mg, 2.4 mmol), $Pd(OAc)_2$ (22.4 mg, 0.10 mmol), $t$$Bu_3PHBF_4$ (58.0 mg, 0.20 mmol), and NaOtBu (384 mg, 4.00 mmol). The reaction mixture was stirred at 120 °C overnight. The reaction mixture was subsequently cooled to room temperature and 20 mL of distilled water was added. The organic layer was extracted three times with 30 mL of ethyl acetate. The combined organic layers were dried over sodium sulfate, and the crude mixture was concentrated under reduced pressure prior to purification by silica gel column chromatography using hexane/dichloromethane = 4/1 as the eluent. **TpIBT-tFFO** was obtained in 70% yield (953 mg, 1.40 mmol).
^{1}H NMR ($CDCl_3$, δ): 8.61 (d, J = 6 Hz, 4H), 8.40 (d, J = 6 Hz, 1H), 7.97 (s, 1H), 7.65 (d, J = 6 Hz, 1H), 7.56–7.52 (m, 4H), 7.47 (d, J = 6 Hz, 1H), 7.39 (t, J = 6 Hz, 4H), 7.25 (t, J = 6 Hz, 1H), 7.14–7.07 (m, 3H), 6.78 (d, J = 6 Hz, 1H), 6.68 (d, J = 12 Hz, 2H), 6.50 (t, J = 12 Hz, 2H), 6.38 (t, J = 6 Hz, 2H), 6.06 (d, J = 12 Hz, 2H), 5.69 (s, 1H), 2.70–2.58 (m, 4H); ^{13}C NMR ($CDCl_3$, δ): 172.68, 170.96, 147.59, 147.50, 146.18, 145.68, 145.58, 144.96, 144.05, 142.36, 136.41, 132.53, 132.05, 131.49, 130.13, 128.85, 128.52, 128.07, 127.66, 127.03, 126.27, 126.08, 125.44, 125.37, 125.33, 125.23, 123.58, 122.49, 120.93, 119.67, 77.22, 77.01, 76.80, 54.77, 46.01, 36.02; APCI-MS (*m/z*): $[M+H]^+$ calcd. for $C_{49}H_{34}N_4$, 679.2856; found, 679.2857; Elemental analysis calcd. (%) for $C_{49}H_{34}N_4$: C, 86.70; H, 5.05; N, 8.25; found: C, 86.78; H, 5.18; N, 8.00.

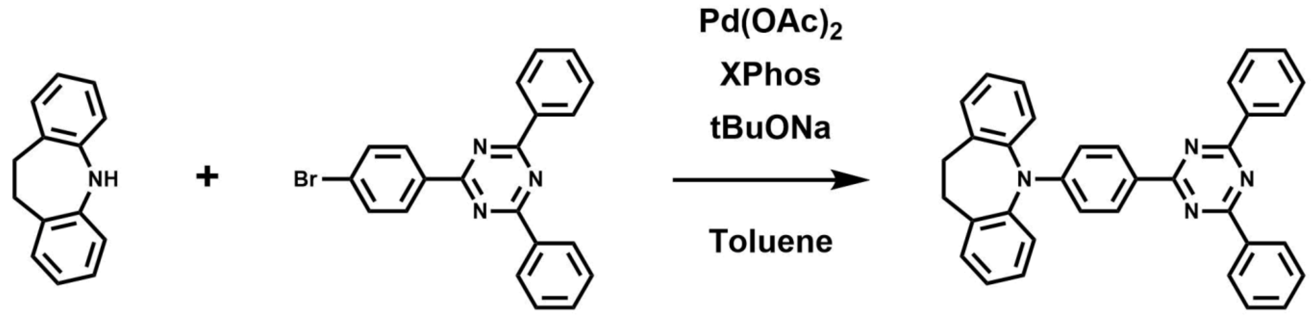

IB-TRZ

IB-TRZ: 25 mL of anhydrous toluene was added to a 50 mL round bottom Schlenk flask containing 10,11-dihydro-5*H*-dibenzo[*b*,*f*]azepine (550 mg, 2.83 mmol), 2-(4-bromophenyl)-4,6-diphenyl-1,3,5-triazine (1.01 g, 2.58 mmol), XPhos (36.9 mg, 0.08 mmol), $Pd(OAc)_2$ (12.1 mg, 0.05 mmol), and *t*BuONa (769 mg, 8.00 mmol). The reaction mixture was stirred at 120 °C overnight. The reaction mixture was subsequently cooled to room temperature and 20 mL of distilled water was added. The organic layer was then extracted three times with 30 mL of ethyl acetate. The combined organic layers were dried over sodium sulfate, and the crude mixture was concentrated under reduced pressure prior to purification by silica gel column chromatography using hexane/dichloromethane = 4/1 as the eluent. **IB-TRZ** was obtained in 41% yield (530 mg, 1.05 mmol).

^{1}H NMR ($CDCl_3$, δ): 8.72 (dd, J = 8.1 Hz, 1.2 Hz 4H), 8.53 (d, J = 9.6 Hz, 2H), 7.58–7.52 (m, 6H), 7.45 (d, J = 6.6 Hz, 2H), 7.31–7.25 (m, 6H), 6.73 (d, J = 9 Hz, 2H), 3.03 (s, 4H); ^{13}C NMR ($CDCl_3$, δ): 171.27, 171.05, 152.56, 142.82, 137.95, 136.67, 132.09, 131.07, 130.46, 129.53, 128.78, 128.49, 127.61, 127.25, 125.29, 112.37, 77.22, 77.01, 76.80, 30.69; ^{15}N NMR ($CDCl_3$, δ): 246.05, 245.33, 99.65; APCI-MS (*m/z*): $[M+H]^+$ calcd. for $C_{35}H_6N_4$, 503.2230; found, 503.2231; Elemental analysis calcd. (%) for $C_{35}H_6N_4$: C, 83.64; H, 5.21; N, 11.15; found: C, 83.85; H, 5.25; N, 10.98.

TpIBT-tFFO : 1H

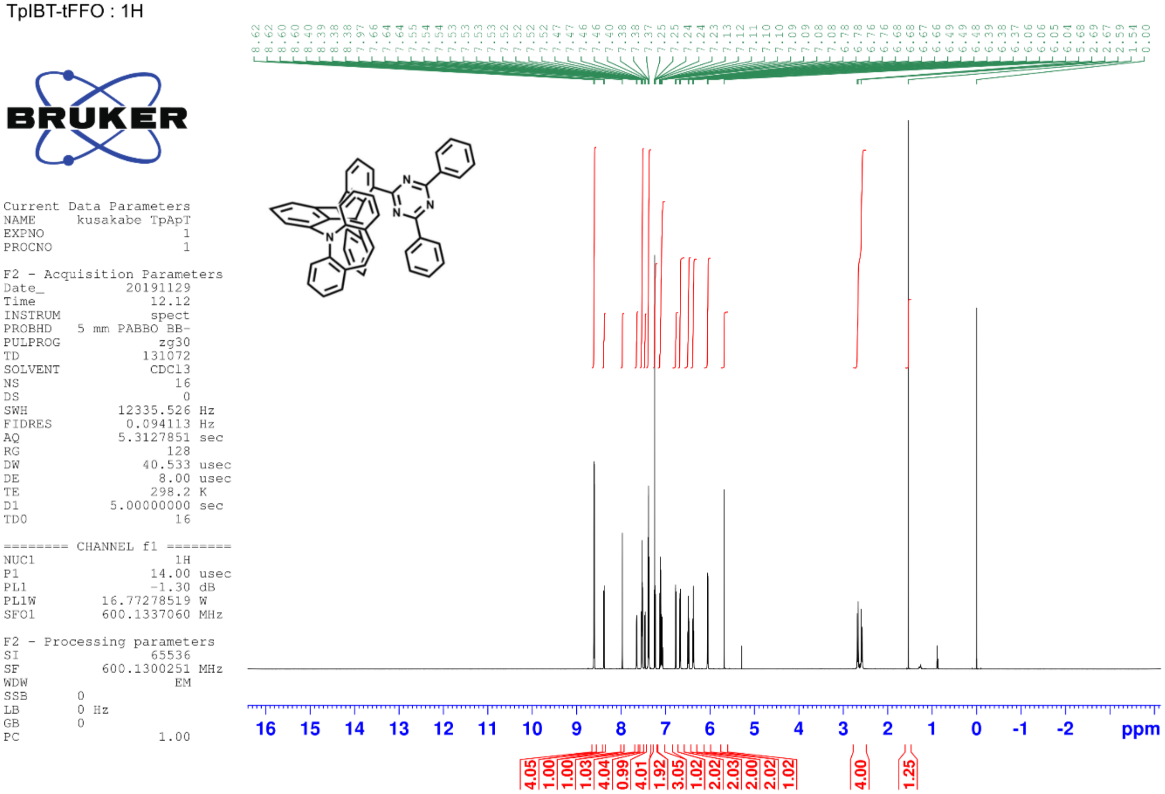

TpIBT-tFFO : 13C

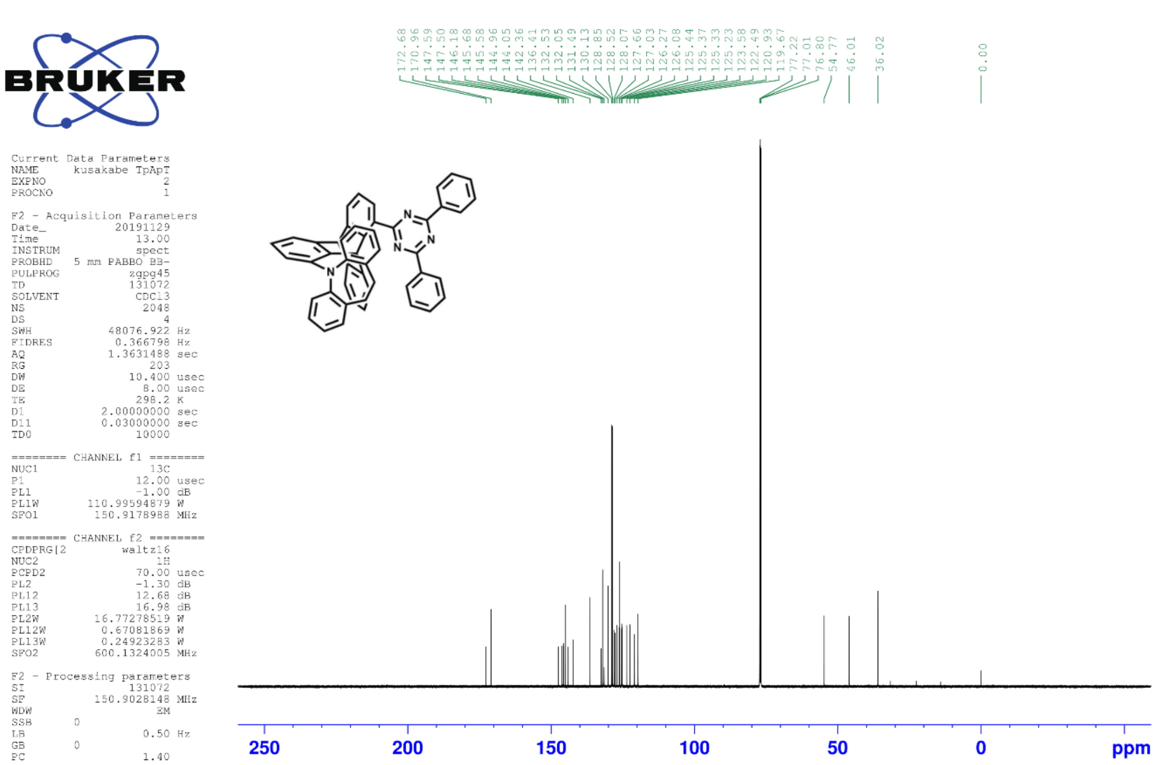

IB-TRZ : 1H

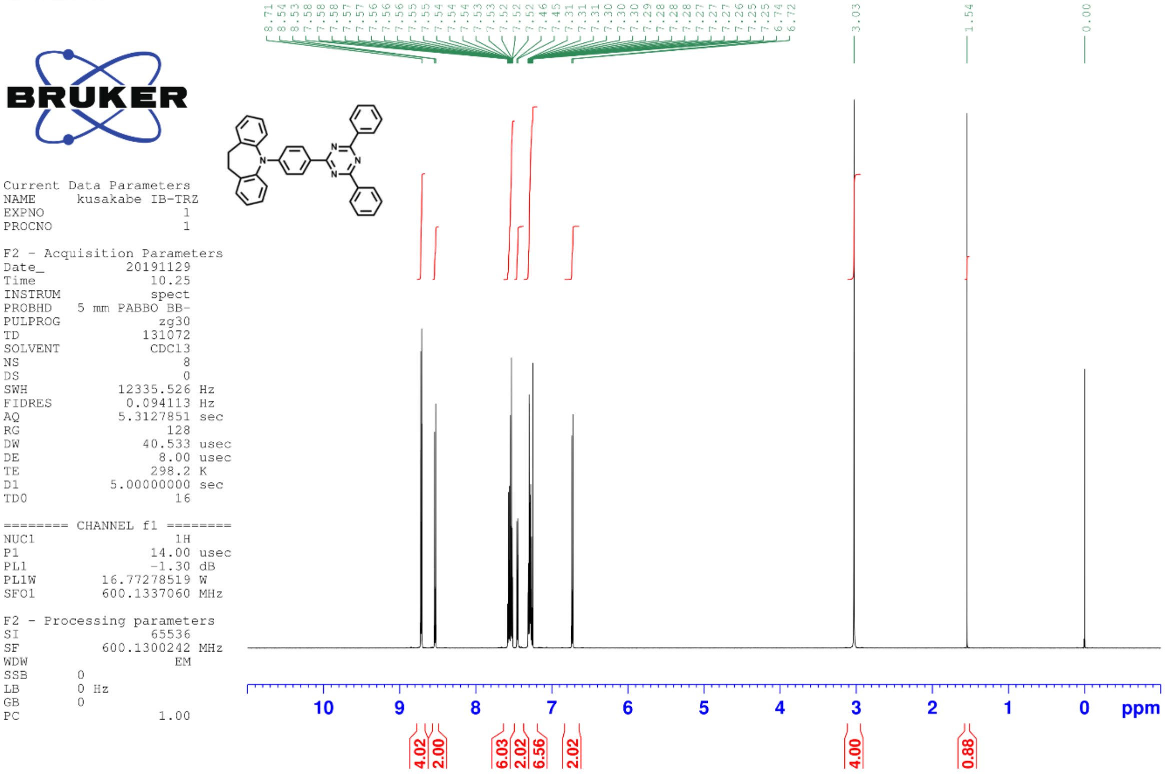

IB-TRZ : 13C

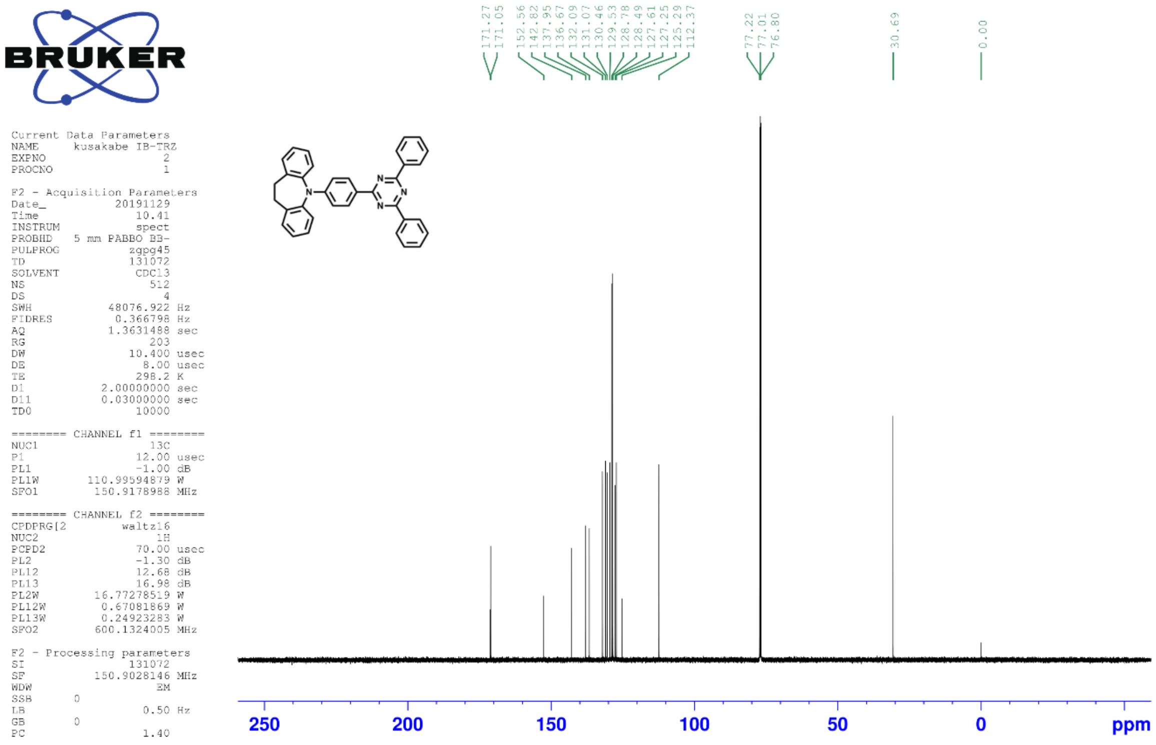

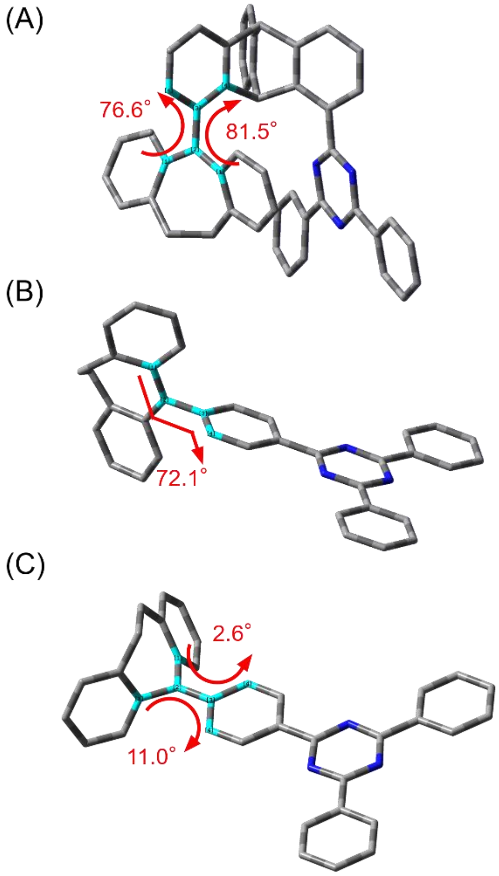

Figure S1. The dihedral angles of the DFT-optimized structures of (A) TpIBT-tFFO, (B) IB-TRZ-E, and (C) IB-TRZ-A.

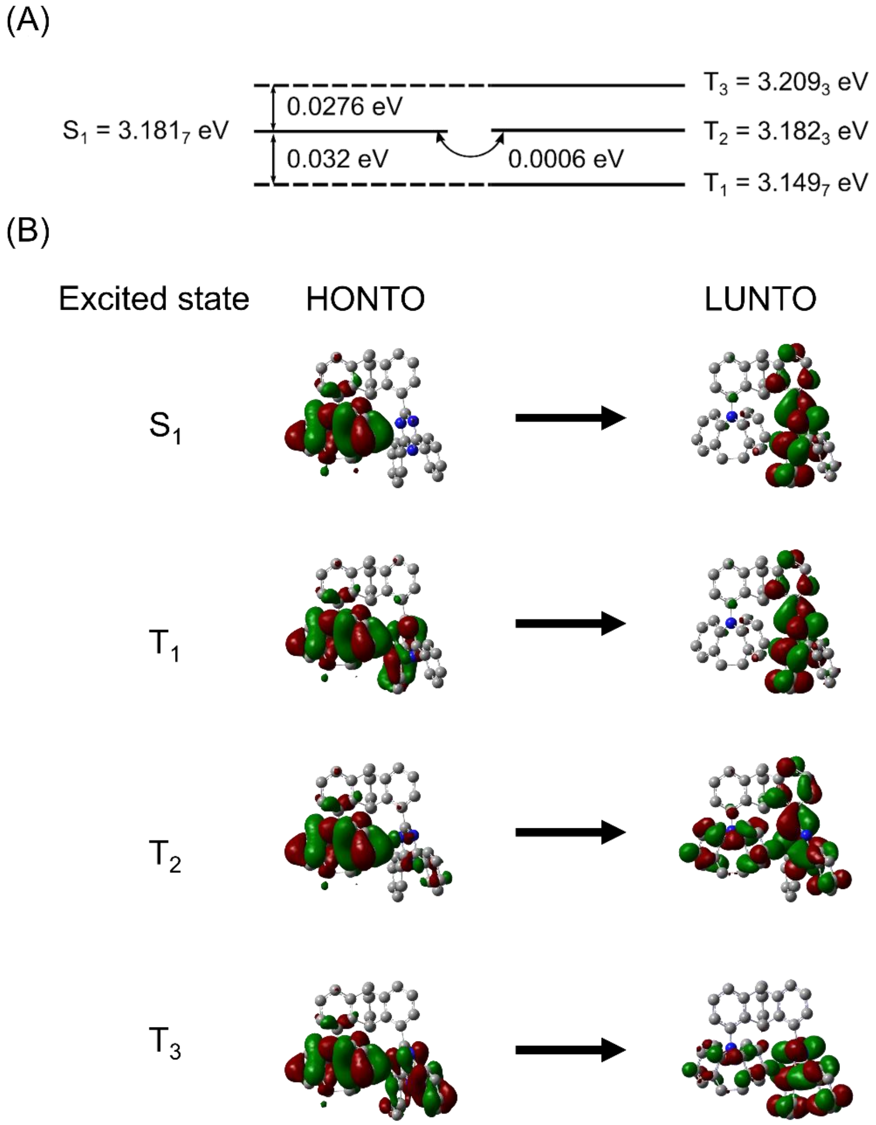

Figure S2. (A) The energy diagram of TpIBT-tFFO. (B) HONTO and LUNTO of each excited state.

Table S1. The SOCMEVs between S_1 and T_1 as well as between S_1 and T_2 calculated at the LCY-ωPBE/TZP level of theory.

Emitter	$S_1 \rightarrow T_1$ (cm^{-1})	$S_1 \rightarrow T_2$ (cm^{-1})
**TpIBT-tFFO**	0.02	0.46

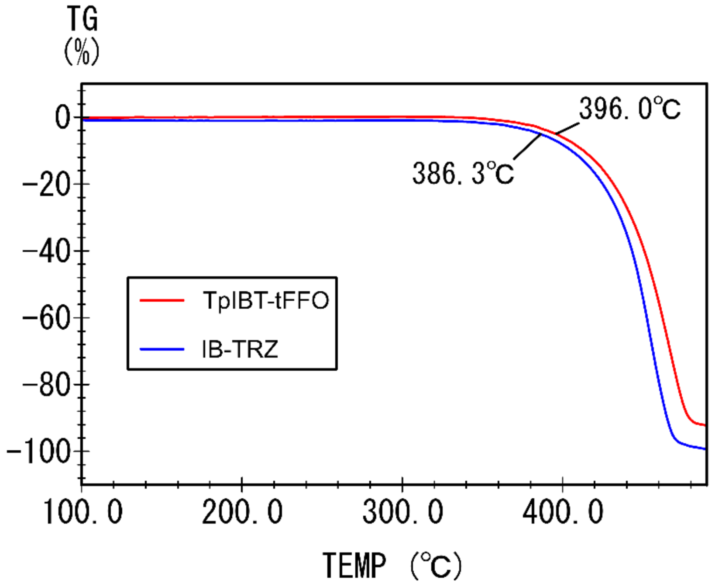

Figure S3. The TGA curves of TpIBT-tFFO (red) and IB-TRZ (blue) at atmospheric pressure under Ar gas flow of 150 mL min^{-1}. The points show 5% weight loss of the TGA curve (decomposition temperature).

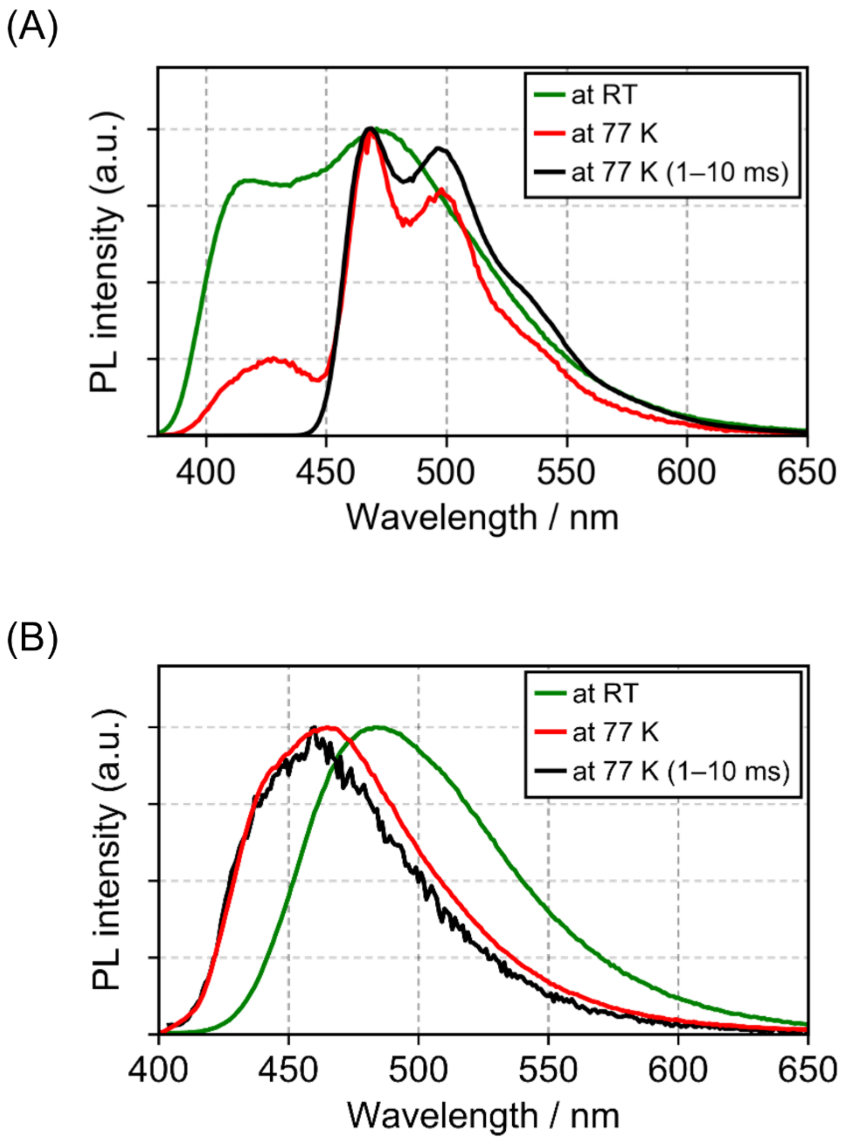

Figure S4. PL spectra at RT (green), 77 K (red), and 77 K in the range of 1–10 ms (black) in a toluene solution for (A) IB-TRZ and (B) TpIBT-tFFO.

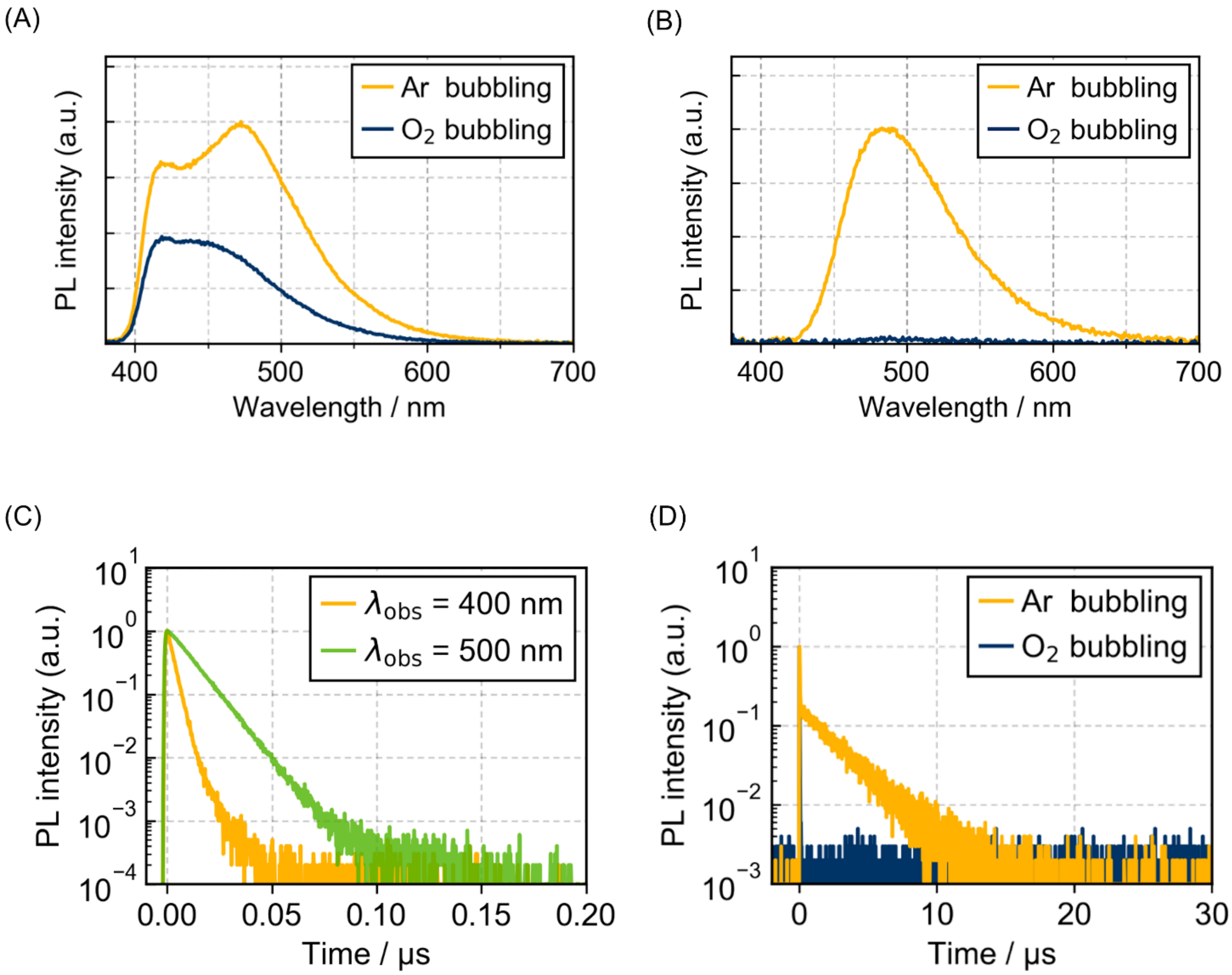

Figure S5. PL spectra in a toluene solution after 15 min of Ar gas bubbling (yellow) and 15 min of O_2 gas bubbling (navy) for (A) IB-TRZ and (B) TpIBT-tFFO. PL decay curves in a toluene solution for (C) IB-TRZ observed at 400 nm (yellow) and 500 nm (yellow green) after 15 min of Ar gas bubbling and (D) TpIBT-tFFO observed at 485 nm after 15 min of Ar gas bubbling (yellow) and 15 min of O_2 gas bubbling (navy).

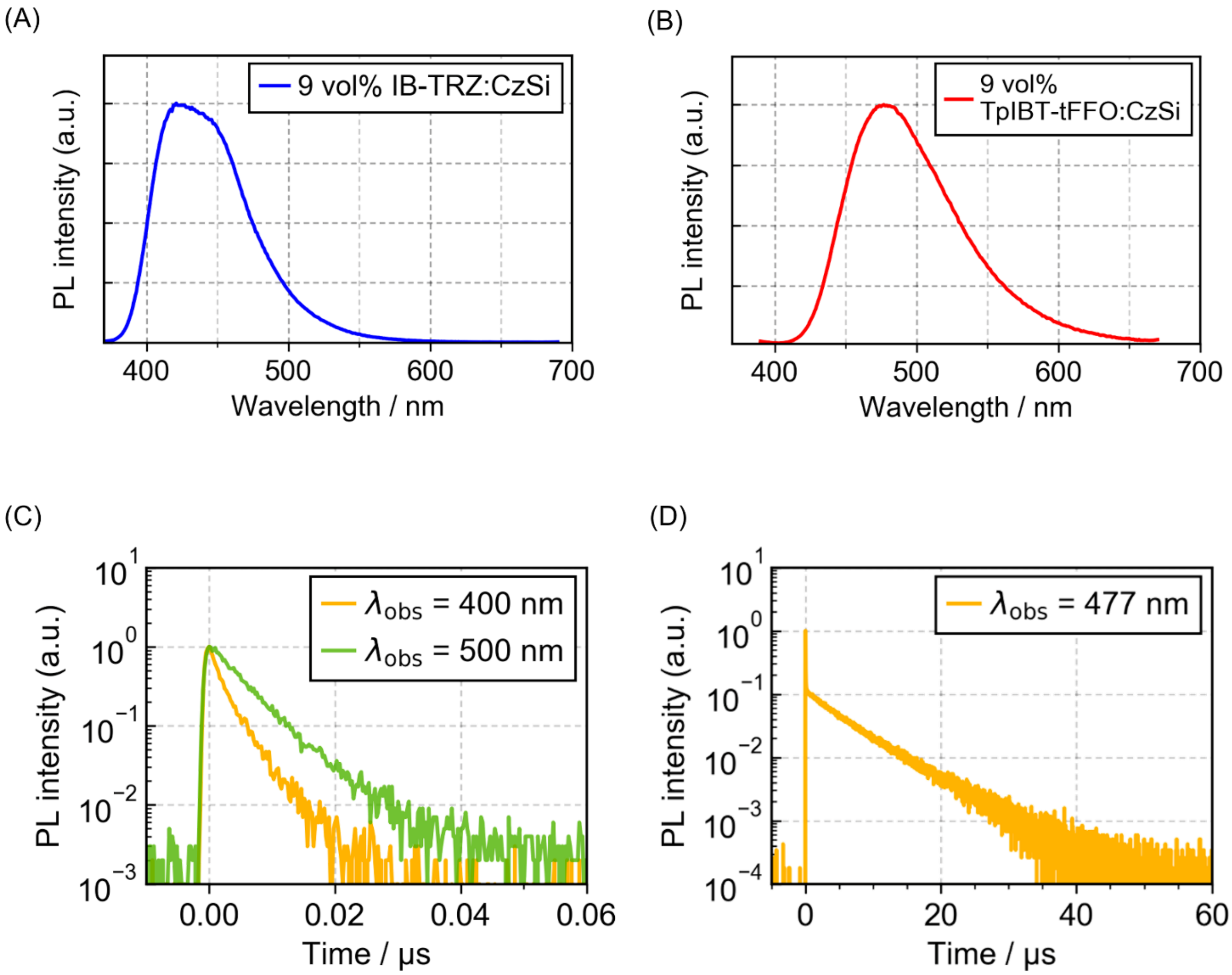

Figure S6. The PL spectra of the 9 vol% emitter:CzSi film for (A) IB-TRZ and (B) TpIBT-tFFO. PL decay curves of the 9 vol% emitter:CzSi film for (C) IB-TRZ observed at 400 nm (yellow) and 500 nm (yellow green), and (D) TpIBT-tFFO observed at 477 nm.

References

Samanta, P. K., Kim, D., Coropceanu, V., and Brédas, J. L. (2017). Up-Conversion intersystem crossing rates in organic emitters for thermally activated delayed fluorescence: impact of the nature of singlet vs triplet excited states. *J. Am. Chem. Soc.* 139, 4042–4051. doi:10.1021/jacs.6b12124

Wada, Y., Nakagawa, H., Matsumoto, S., Wakisaka, Y., and Kaji, H. (2020). Organic light emitters exhibiting very fast reverse intersystem crossing. *Nat. Photon.* doi:10.1038/s41566-020-0667-0
